# A novel camptothecin derivative, ZBH-01, exhibits superior antitumor efficacy than irinotecan by regulating the cell cycle

**DOI:** 10.1186/s12967-023-04196-2

**Published:** 2023-06-29

**Authors:** Yongqi Li, Dawei Zhao, Wenqiu Zhang, Miaomiao Yang, Zhihui Wu, Weiguo Shi, Shijie Lan, Zhen Guo, Hong Yu, Di Wu

**Affiliations:** 1 Department of Cancer Centre, The First Hospital of Jilin University, Changchun, 130021 China; 2 Institute of Translational Medicine, The First Hospital of Jilin University, Changchun, 130061 China; 3 Department of Breast Tumor, Jilin Cancer Hospital, Changchun, 130012 China; 4 Institute of Pharmacology and Toxicology Academy of Military Medical Sciences, Beijing, 100850 China; 5 Cell Biology Laboratory, Jilin Province Institute of Cancer Prevention and Treatment, Jilin Cancer Hospital, Changchun, 130012 China

**Keywords:** Irinotecan derivative ZBH-01, Colorectal cancer, Next-generation sequencing, Differentially expressed genes, Cell cycle, Apoptosis

## Abstract

**Background:**

Irinotecan (CPT-11) is a classic chemotherapeutic agent that plays an important role in the clinical treatment of metastatic colon cancer and other malignant tumors. We previously designed a series of novel irinotecan derivatives. In this study, we select one representative, ZBH-01, to investigate its sophisticated antitumor mechanism in colon tumor cells.

**Methods:**

The cytotoxic activity of ZBH-01 on colon cancer cells was evaluate by MTT or Cell Counting Kit-8 (CCK8) assay, 3D and xenograft model. The inhibitory effect of ZBH-01 on TOP1 was detected by DNA relaxation assay and Immuno Complex of Ezyme (ICE) bioassay. The molecular mechanism of ZBH-01 was explored by Next-Generation Sequencing (NGS), bioinformatics analyses, flow cytometry, qRT-PCR, and western blot etc.

**Results:**

ZBH-01 can induce obvious DNA damage and has superior antitumor activity against colon cancer cells compared to CPT-11 and SN38 (7-Ethyl-10-hydroxy camptothecin, the in vivo active form of CPT-11) both in vivo and in vitro. Its inhibitory effect on topoisomerase I (TOP1) was also comparable with these two control drugs. There are a much larger number of 842 downregulated and 927 upregulated mRNAs in ZBH-01 treatment group than that in the controls. The most significantly enriched KEGG pathways for these dysregulated mRNAs were DNA replication, the p53 signaling pathway, and the cell cycle. After constructing a protein–protein interaction (PPI) network and screening out a prominent cluster, 14 involved in the cell cycle process was identified. Consistently, ZBH-01 induced G_0_/G_1_ phase arrest in colon cancer cells, while CPT-11/SN38 caused S phase arrest. The initiation of apoptosis by ZBH-01 was also superior to CPT-11/SN38, followed by the increased expression of Bax, active caspase 3, and cleaved-PARP, and decreased expression of Bcl-2. Additionally, CCNA2 (cyclin A2), CDK2 (cyclin-dependent kinase 2), and MYBL2 (MYB proto-oncogene like 2) might be involved in the G_0_/G_1_ cell cycle arrest induced by ZBH-01.

**Conclusions:**

ZBH-01 can be an antitumor candidate drug for preclinical study in the future.

## Background

Colorectal cancer (CRC) is one of the main five cancer types and the five most common causes of cancer-related deaths in China, with the incidence and mortality are continuously increasing [1, 2]. Despite significant progress in precision medicine, Irinotecan (CPT-11) remains the primary chemotherapeutic agent for the treatment of metastatic CRC [3, 4].

CPT-11 is a camptothecin (CPT) analog, which was discovered from plant extracts more than 60 years ago. It disturbs the catalytic cycle of DNA TOP1 by stabilizing the reversible covalent enzyme–DNA cleavable complex. Moreover, by forming a drug-enzyme–DNA ternary complex during DNA synthesis, CPT-11 triggers the formation of irreversible single-stranded DNA break when the cleavable complex collides with the DNA replication fork [5]. This specific cytotoxic effect characterizes CPT-11 as a potent antitumor agent. However, its poor water solubility and serious side effects hinder its clinical applications. Until now, only two CPT analogs (irinotecan and topotecan) have been approved for cancer treatment worldwide [6–8]. A breakthrough in the development of novel CPT analogs is still an urgent problem to be solved.

In the last 10 years, our group designed, synthesized, and characterized a series of novel irinotecan derivatives with higher antitumor activity and lower toxicity compared to CPT-11 [9–12]. However, their molecular mechanisms need to be precisely elucidated. This study explored the mechanism of one representative, ZBH-01, which showed a higher inhibitory effect on the development of colon tumors than CPT-11 preliminarily.

## Materials and methods

### Agents

**Fig. 1:**
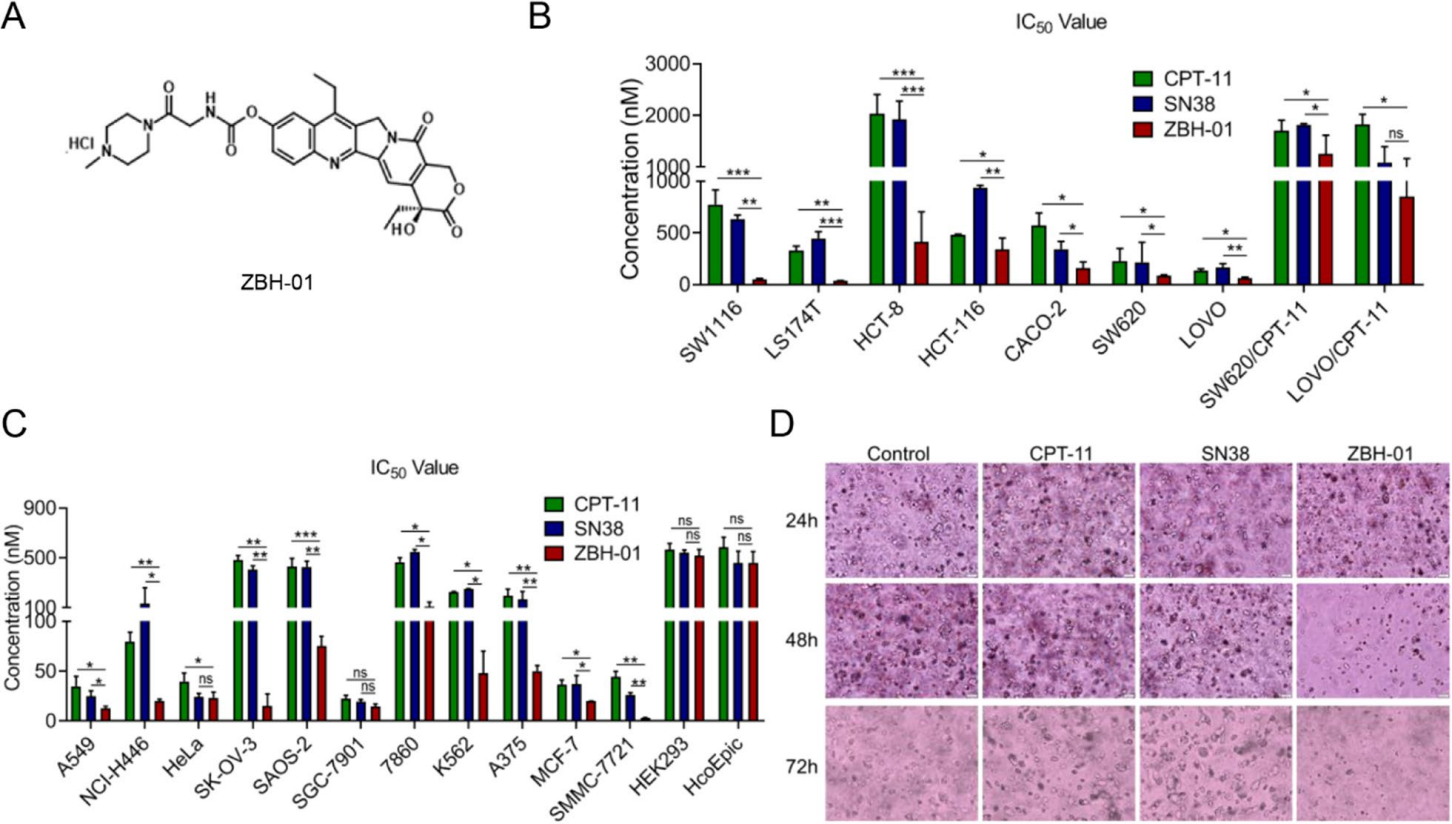
Comparison of the anti-proliferation effects of ZBH-01, CPT-11, and SN38. **A** Chemical structural of ZBH-01. **B** The IC_50_ values of ZBH-01, CPT-11, and SN38 in colorectal tumor cells and SW620/CPT-11 and LOVO/CPT-11 cells evaluated by MTT or CCK8 assay. **C** The IC_50_ values of ZBH-01, CPT-11, and SN38 in non-colorectal tumor cells and HcoEpic and 293 cells evaluated by MTT or CCK8 assay. **D** The anti-tumorigenesis effects of ZBH-01, CPT-11, and SN38 in LS174T cells evaluated by 3D cell culture. The representative Images were captured by a fluorescence microscope (200 ×). ANOVA & two-tailed t-test, n = 3. *p < 0.05, **p < 0.01, ***p < 0.001. SW1116, LS174T, HCT-8, HCT-116, CACO-2, SW620 and LOVO, colon adenocarcinoma. A549 and NCI-H446, non-small cell lung cancer. HeLa, cervical carcinoma. SK-OV-3, ovarian cancer. SAOS-2, osteosarcoma. SGC-7901, gastric adenocarcinoma. 7860, renal carcinoma. K562, chronic myelogenous leukemia. A375, melanoma. MCF-7, breast cancer. SMMC-7721, hepatoma. HcoEpic, human colon mucosal epithelia. HEK293, human embryonic kidney cell

ZBH-01 (Fig. 1A) was synthesized by the Institute of Pharmacology and Toxicology Academy of Military Medical Sciences (China). CPT-11 and SN38 (the activated form of irinotecan in vivo) were provided by the same institution. All chemical agents were dissolved in dimethyl sulfoxide (DMSO; Sigma-Aldrich, St. Louis, MO, USA) and stored at −80 ℃ before use.

### Cell lines

Eighteen human cancer cell lines including seven colon cancer cell lines and two CPT-11-resistant colorectal cancer cell lines SW620/CPT-11 and LOVO/CPT-11, a normal human colon mucosal epithelial cell lines (HcoEpic), and the human HEK293 cell lines were maintained in DMDM or RPMI 1640 media (Life Technologies, Grand Island, NY, USA) supplemented with 10% fetal bovine serum (FBS, Gibco), 2 mmol/l glutamine, 100 U/ml penicillin, and 100 µg/ml streptomycin in a humidified incubator at 37 ℃ and 5% CO_2_.

### *Cell viability assay *in vitro

The cytotoxic activity of ZBH-01 was evaluated against the above cell lines using MTT or Cell Counting Kit-8 (CCK8) assay as previously described [9, 11]. HEK293 and HcoEpic cells were used as control cells. The different cell lines, including the colon cancer cell lines, were treated with different concentrations of ZBH-01, CPT-11, and SN38. After 72 h of incubation, the absorbance of cells in each group was measured at 450 nm using a spectrophotometer. The IC_50_ values (50% inhibition of cell growth) were calculated by Statistical Product and Service Solutions (SPSS) 23.0.

### 3D culture

Hydrogel (The Well Bioscience, Shanghai, China) was mixed with cell culture medium to form a hydrogel matrix with the dilution ratio 1:2 v/v. Then the hydrogel matrix and LS174T cells were uniformly mixed and were seeded into a six-well plate at 1 × 10^6^ cells/ml. The medium was added to cover the hydrogel carefully. Afterwards, cells were treated with 50 nmol/L ZBH-01, CPT-11, and SN38 for 24–72 h, respectively. Images were captured using a fluorescence microscope (Olympus IX51, Tokyo, Japan).

### DNA relaxation assay

The DNA relaxation assay was performed according to the manufacturer's instructions (TopoGEN, Inc., Port Orange, FL, USA) [11]. The reaction was conducted in a dosage-dependent manner. We also performed qRT-PCR and western blot to evaluate the inhibition of ZBH-01 on TOP1 in LS174T or SW1116 colon cancer cells. The primers of TOP1 (DHS626257) were provided by XYbiotech (Shanghai, China). The forward (5'‑CTACCTCATGAAGATCCTCACCGA‑3') and reverse (5'‑TTCTCCTTAATGTCACGCACGATT‑3') primers of β‑actin were provided by Shanghai Generay Biotech Co., Ltd. Total RNA isolation, first-strand cDNA synthesis, and the qRT-PCR assay was performed as described below (see qRT-PCR assay section) [13].

### Immuno complex of ezyme (ICE) bioassay

To detect the alteration of the covalent TOP1-DNA complex in colon cancer cells after ZBH-01 treatment, the TOP1-DNA adducts were isolated by the in vivo complex of enzyme (ICE) bioassay [14]. Briefly, 1 × 10^6^ treated or untreated LS174T cells were lysed with 1 ml of 1% sarkosyl. After homogenization by a Dounce homogenizer, cell lysates were gently layered on four step gradients CsCl solutions with densities of 1.82, 1.72, 1.50, and 1.45 (2 ml of each). Then the tubes were centrifuged at 165,000 g for 24 h at 20 ℃. Next, half-milliliter fractions were collected from the bottom of the tubes. Aliquots of each fraction (100 ml) were diluted with an equal volume of 25 mM sodium phosphate buffer (pH 6.5) and applied to Immobilon-P membranes (Millipore) by a slot-blot vacuum manifold. The topoisomerase-DNA adducts were detected by western blot with a anti-TOP1 mAb.

### next-generation sequencing (NGS)

First, LS174T cells were subjected to 50 nmol/L ZBH-01, CPT-11, and SN38 for 24 h. Total RNA was extracted using TRIzol reagent (Invitrogen, CA, USA) following the manufacturer’s instructions. Total RNA quantity and purity were analyzed by Bioanalyzer 2100 and RNA 6000 Nano Lab Chip Kit (Agilent, CA, USA) with RIN number > 7.0. Then, 3 μg RNA per sample was used to prepare RNA-Seq libraries. Sequencing libraries were generated using NEBNext Ultra II RNA Library Prep Kit for Illumina (NEB, E7760) following the manufacturer’s recommendations. The PCR was performed with Phusion High-Fidelity DNA polymerase, universal PCR primers, and index (X) Primer. Finally, PCR products were purified (AMPure XP system) and library quality was assessed on the Agilent Bioanalyzer 2100 system. Paired-end sequencing was performed using Illumina HiSeq X Ten (Illumina, San Diego, CA). Raw data (raw reads) in the fastq format were processed by the Fastp software. All downstream analyses were based on clean high-quality data. Paired-end clean reads were aligned to the reference genome using STAR V20201. Cufflinks v2.2.1 was used to count the number of reads mapped to each gene. The FPKM of each gene was calculated based on the length of the gene and the count of reads mapped to this gene. The resulting *p-*values were adjusted using Benjamini and Hochberg’s approach for controlling the false discovery rate (FDR).

### Differential expression analysis of mRNAs

To compare mRNA expression differences among ZBH-01, CPT-11, and SN38 groups, a *p*-value < 0.05 and |log_2_ fold change|> 1 were set as the threshold for significantly differential expressions. First, the gene expression profiles of the three groups were compared to the control group. Then the three datasets obtained were analyzed together to obtain commonly expressed genes and a ZBH-01 group-specific differentially expressed gene list.

### Bioinformatics analyses

Gene Ontology (GO) classifications (biological process, cellular component, and molecular function) and the Kyoto Encyclopedia of Genes and Genomes (KEGG) pathway enrichment analyses were hierarchically investigated using ‘clusterProfiler’ in R 3.4.0 (R Foundation, Vienna, Austria) [15] and KEGG Pathway Database (http://www.genome.jp/kegg/pathway.html). The proteins encoded by upregulated and downregulated differentially expressed mRNAs (DEmRNAs) in the ZBH-01 group were used to construct a PPI network using STRING (https://string-db.org/) and Cytoscape 3.5.1 (http://www.cytoscape.org/, The Cytoscape Consortium, San Diego, CA, USA) [16, 17]. We used Cytotype MCODE to identify key network modules and select hub genes.

### qRT-PCR assay

The PCR primers of some genes are shown in Table 1. Besides, the primers of other genes were provided by XYbiotech (Shanghai, China) including BUB1 (catalog number. DHS322648), BUB1B (DHS646748), CCNA2 (DHS819739), CDC20 (DHS889533), CDC25C (DHS740393), CDC45 (DHS793726), CDC7 (DHS095090), CDK2 (DHS278875), CHEK1 (DHS670983), E2F8 (DHS048256), EZF2 (DHS068552), FOXM1 (DHS718140), MCM3 (DHS061267), MCM7 (DHS794915), MYBL2 (DHS516386), ORC1 (DHS500377), PKMYT1 (DHS687810), RAD54L (DHS609809), TOP2A (DHS036498), and TTK (DHS713780). Total RNA isolation was performed with the EasyPure RNA kit (Transgen) according to the manufacturer’s guidelines. RNA concentrations were measured by a microplate reader (BioTek Synergy H1). The TransScript All-in-One First-Strand cDNA Synthesis SuperMix for qPCR kit was used for reverse transcription according to the manufacturer’s instructions. The 20 μL reaction volumes contained 1 μg RNA prepared by combining 4 μL transcript all-in-one supermix for qPCR, 1 μL gDNA remover, and variable RNase-free water. The reverse transcription reactions were performed for 15 min at 42 ℃ and 5 s at 85 ℃. Each reverse transcription product was diluted 10 times by adding 180 μL H_2_O to 20 μL cDNA. The qPCR with TransStart Tip Green qPCR SuperMix kit (Transgen) was also performed according to the manufacturer’s protocol. Briefly, 20 μL reaction volume were prepared by combining 10 μL transstart tip green qPCR supermix, 0.4 μL passive reference dye, 0.5 μL forward primer and 0.5 μL reverse primer, 3 μL cDNA and 5.6 μL ddH_2_O. The cycling conditions were: 94 ℃ for 30 s, 40 cycles at 94 ℃ for 5 s, and 60 ℃ for 30 s. The relative gene expression data were analyzed by the 2^−△△CT^ method.

**Table 1:** Primers for qRT-PCR

Genes	Forward primer (5′-3′)	Reverse primer (5′-3′)
MCL1	TCTCATTTCTTTTGGTGCCTTTG	TAACTAGCCAGTC(TGTTTTGTC
XIAP	GTGAATGCTCAGAAAGACAGTATGC	TGTCCACAAGGAACAAAAACGATAG
BIRC3	GATGCTGGATAACTGGAAAAGAG	TGAAGAAGGAAAAGTAGGCTGAG
MDMX	ATTTTCCTTTTCAGGTATGGC	AGGTACTGTTTTCGTTGTTGG
MDM2	AAGGGAAGAAACCCAAGACAAAGA	GCACATGTAAAGCAGGCCATAAGA
CYC	TGATGCCTTTGTTCTTATTGG	TTTATTATGAAGTGTTCCCAGTG
APAF-1	ATCTGGGCTTCTGATGAAACTGC	CAACACCCAAGAGTCCCAAACAT
CASP9	AGCCAACCCTAGAAAACCTTACC	TCACCAAATCCTCCAGAACCAAT
cBid	GTCACACGCCGTCCTTGCT	CTGTCCGTTCAGTCCATCCCATT
BAX	AGGATGCGTCCACCAAGAAGC	GGCAAAGTAGAAAAGGGCGACA
BCL-XL	GAGAATCACTAACCAGAGACGAGA	GGAGAGAAAGTCAACCACCAGC
PTEN	TAAGGACCAGAGACAAAAAGGGA	GGCAGACCACAAACTGAGGATT
CDK6	TGATCAACTAGGAAAAATCTTGGAC	GGCAACATCTCTAGGCCAGT
NEK2	TGTCTCTGGCAAGTAATCCAG	CAGGTCCTTGCACTTGGACT
CCND1	AGCTGTGCATCTACACCGAC	TGTGAGGCGGTAGTAGGACA
CCNE2	ACCTCATTATTCATTGCTTCCAA	TCTTCACTGCAAGCACCATC
BIRC5	TTCTCAGTGGGGCAGTGGATG	TTTCTCAAGGACCACCGCATCT
CDK4	CTTCTGCAGTCCACATATGCAACA	CAACTGGTCGGCTTCAGAGTTTC
P53	AGCTTTGAGGTGCGTGTTTGTG	TCTCCATCCAGTGGTTTCTTCTTTG
RB1	CACAACCCAGCAGTTCAATATC	TGAGATCACCAGATCATCTTCC
ATM	TGTGACTTTTCAGGGGATTTG	ATAGGAATCAGGGCTTTTGGA
ATR	GGGAATCACGACTCGCTGAA	CTAGTAGCATAGCTCGACCATGGA
XAF1	GCCTACTTGCTGTGGTGGTCTTGT	ACGCCTGGTTTGTTGAGGGTTTT
P21	TTAAACAAAAACTAGGCGGTTGA	AGGAGAACACGGGATGAGGA
CASP3	TGGCATTGAGACAGACA	GGCACAAAGCGACTG

### Flow cytometry

To analyze the changes in the cell cycle, cell apoptosis, and mitochondrial membrane potential (MMP), SW1116 and LS174T colon cancer cells were harvested after exposure to 50 nmol/L ZBH-01, CPT-11, and SN38 for 12–72 h. Then, cells were stained by FxCycle^™^ PI/RNase Staining Solution (Invitrogen), FITC Annexin V Apoptosis Detection kit I (BD Biosciences, San Diego, CA, USA), and Mitochondrial Membrane Potential Detection Kit (BD Biosciences, San Diego, CA, USA) according to the manufacturer's protocol. Flow cytometry was performed using a FACS Calibur system (BD Dickinson). A minimum of 10,000 events was recorded for each sample. Data were acquired and analyzed by CellQuest (BD Biosciences), ModFit 4.0 (BD Biosciences), and FlowJo (TreeStar, Inc., Ashland, OR) software [11].

### Western blot

First, SW1116 and LS174T cells were seeded in 6-well plates with a complete medium as previously described [9]. After cultivation for 24 h, cells were treated with 50 nmol/L ZBH-01, CPT-11, and SN38 for 48 h and harvested. Next, cell pellets were lysed and the protein concentrations of each sample were measured. Then, samples were separated by 10% SDS-PAGE and transferred to a polyvinylidene difluoride membrane (GE Healthcare, Piscataway, NJ). Samples were incubated with corresponding primary antibodies (1:500–1000 dilution) in 5% BSA at 4 ℃ overnight. Then, the membrane was washed and incubated with appropriate secondary antibodies at room temperature for 1 h. The antibodies included TOP1, caspase 3, PARP, P53, Bax, Bcl-xL (54H6), p-BRCA1, p-H2AX, p-CHK1, p-CHK2, p-P53 (Cell Signaling Technology, Shanghai, China). β-actin was used as the internal reference (1:5000 dilution). Protein bands were visualized with ECLplus Western Blotting Detection Reagents (GE Healthcare) on the ECL System (Millipore, Billerica, MA) [11].

### *Antitumor activity of ZBH-01 *in vivo

The antitumor activity in vivo was evaluated using 6–8 week-old female nude mice as previously described [10] in accordance with the Aimal Eperimental Gidelines of Jilin University. The xenografts model was established by subcutaneous injection of 1 × 10^6^ LS174T cells in the left flank. Tumor growths were measured with vernier calipers twice a week. When masses reached 100–150 mm^3^, mice were randomly assigned to the treatment (n = 6) or control (n = 6, treated with saline) groups. Mice in the treatment group intravenously (i.v.) received ZBH-01 (40 mg/kg) or CPT-11 (40 mg/kg) using the q4dx3w schedule [18, 19]. Animals were treated for 21 d, monitored twice a week for signs of toxicity, and weighed every 3 d. At the end of the experiment, all mice were euthanized. Their main organs such as the heart, liver, spleen, lung, kidney and tumor tissues were stripped. Tissue sections were prepared and stained with hematoxylin and eosin (H&E) according to standard protocol. Tumor volume and growth inhibition were calculated [20]. Tumor volume (TV) = length × width^2^ /2; Relative tumor volume (RTV) = TV_t_ /TV_0_ (TV_t_: tumor volume at day t, TV_0_: tumor volume at the initiation of treatment); Relative tumor proliferation (T/C) = RTV_t_ /RTV_C_ × 100% (RTV_t_: the treatment group RTV, RTV_C_: the control group RTV). To detect tumor cell apoptosis induced by ZBH-01 in vivo, fresh tumor tissues were dissected into small pieces and crushed by the flat end of the plunger of sterile syringe. Cell suspensions were then filtered and centrifuged for 10 min 1200 g, 4 ℃. Afterwards, each sample was stained by FITC-Annexin V/PI as described in the previous Sect. “Flow cytometry”.

### Statistical analyses

Data are expressed as means ± standard deviations (SDs). One-way analysis of variance (ANOVA), χ^2^ test, the two-tailed Student’s *t-*test, or Mann–Whitney *U* test was performed using SPSS 23.0 software (SPSS, Inc., Chicago, IL, USA) and GraphPad Prism version 8.01 (GraphPad Software, San Diego, CA, USA). A *p* < 0.05 was considered statistically significant.

## Results

### ZBH-01 showed superior antitumor effects than CPT-11 and SN38

CPT-11 is a prodrug and SN38 represents its metabolically-activated form in vivo. Therefore, we used CPT-11 or SN38 as positive controls in our study. We evaluate the anti-proliferation effect of ZBH-01 on multiple tumor cell lines derived from different origins. The results showed that the IC_50_ values of ZBH-01 are significantly lower than that of CPT-11 and SN38 in most tumor cell lines, even in the two CPT-11 resistant colorectal cancer cell lines SW620/CPT-11 and LOVO/CPT-11 (Fig. 1B, C). More prominently, the IC_50_ value of ZBH-01 is comparable with that of CPT-11 and SN38 in normal epithelial HcoEpic cells and 293 cells. In addition, the result of 3D cell culture further verified the superior anti-tumorigenesis effect of ZBH-01 (Fig. 1D). Altogether. ZBH-01 showed higher anti-proliferative effect as compare to CPT-11 and SN38. We selected LS174T and SW1116 cells for subsequent experiments. The IC_50_ value of ZBH-01 on the two cell lines is about 50 nmol/L.

### The reduction of TOP1 activity by ZBH-01 is weaker compared to CPT-11 and SN38

**Fig. 2:**
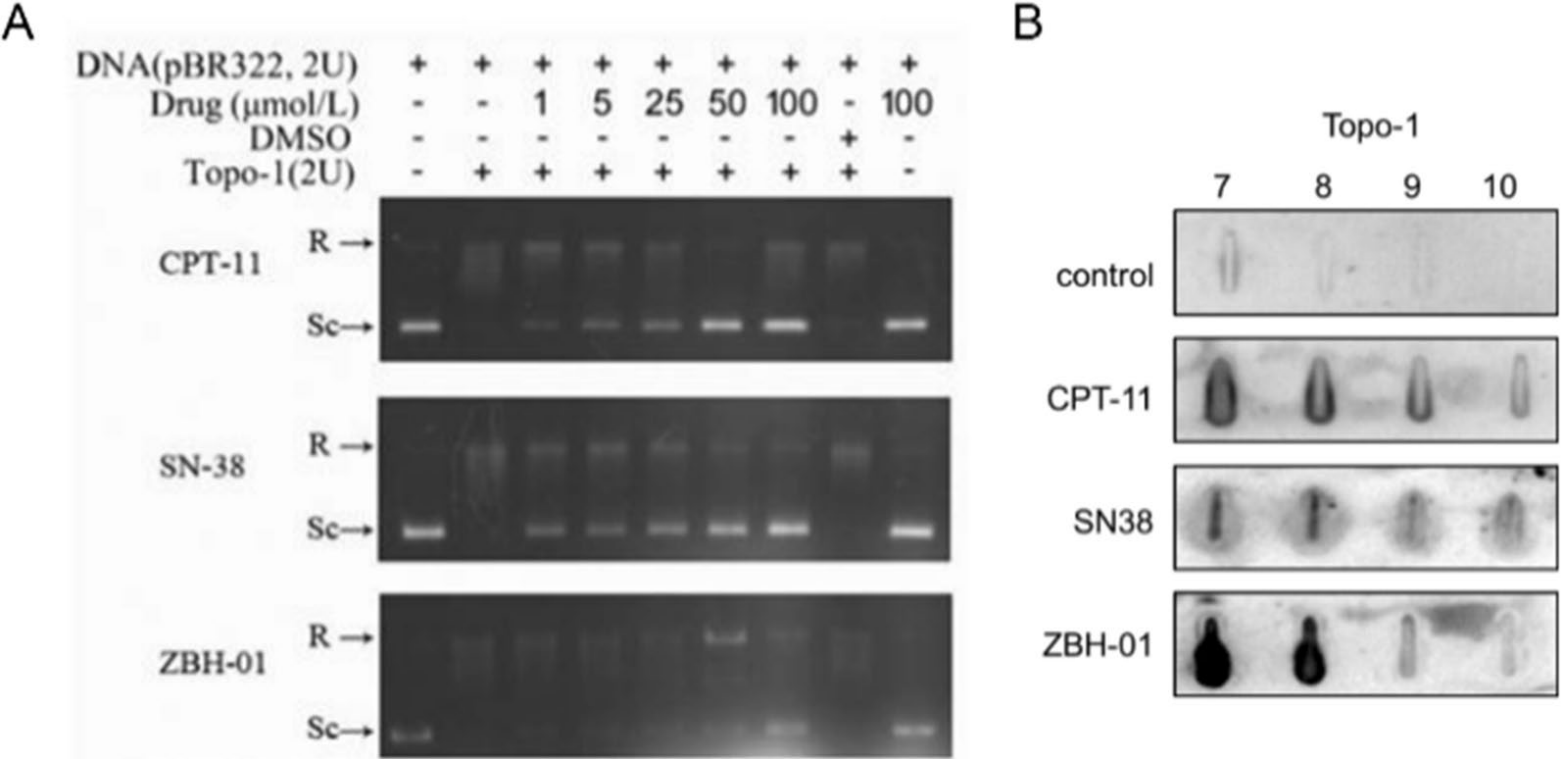
Inhibitory effect of ZBH-01 on TOP1. **A** TOP1-catalyzed DNA relaxation was inhibited by CPT-11, SN-38, and ZBH-01. The DNA strand breakage induced by TOP1 was evaluated by the conversion of the double-stranded supercoiled DNA to a relaxed form. The position of supercoiled DNA (Sc) and relaxed DNA (R) are indicated. **B** The image of immunoblot showed the signals of TOP1 band in the DNA-containing fractions in LS174T cells after treatment with ZBH-01, CPT-11, and SN38. The fractions are indicated by numbers

By forming a drug–enzyme–DNA complex, the drug prevents the relegation step normally catalyzed by topoisomerase, finally inhibiting the relaxation activity. Hence, to explore the interaction of ZBH-01 and TOP1, we performed a DNA relaxation assay. The inhibitory effect of ZBH-01 on TOP1 was weaker than CPT-11 and SN38 (Fig. 2A). This result was contradictory to our previous reports [9, 11], which might indicate the heterogeneity and complexity of ZBH-01 mechanisms. Nevertheless, after treatment with 50 nmol/L ZBH-01 for 24 h, LS174T cells presented decreased protein levels of TOP1 (Fig. 9), while its mRNA levels were not suppressed (Fig. 5). This result was consistent with previous studies, indicating a significant downregulation of TOP1 in tumor cells treated with topoisomerase inhibitors [19, 20]. The reasons for our conflicting results remain to be further elucidated.

ZBH-01 mediated TOP1 trapping in LS174T cells was measured by the ICE bioassay, which can detect the covalently link of TOP1 to genomic DNA after drug treatment. As expected, the result of immunoblot showed that the signals of TOP1 band in the DNA-containing fractions in ZBH-01-treated cells were more enhanced than that in CPT-11- and SN38-treated cells (Fig. 2B). This result further verify the inhibitory ability of ZBH-01 to TOP1 through chromatin-associated TOP cleavage complexes.

### ZBH-01 treatment leads to the highest number of DEmRNAs in LS174T cells compared to CPT-11 and SN38

**Fig. 3:**
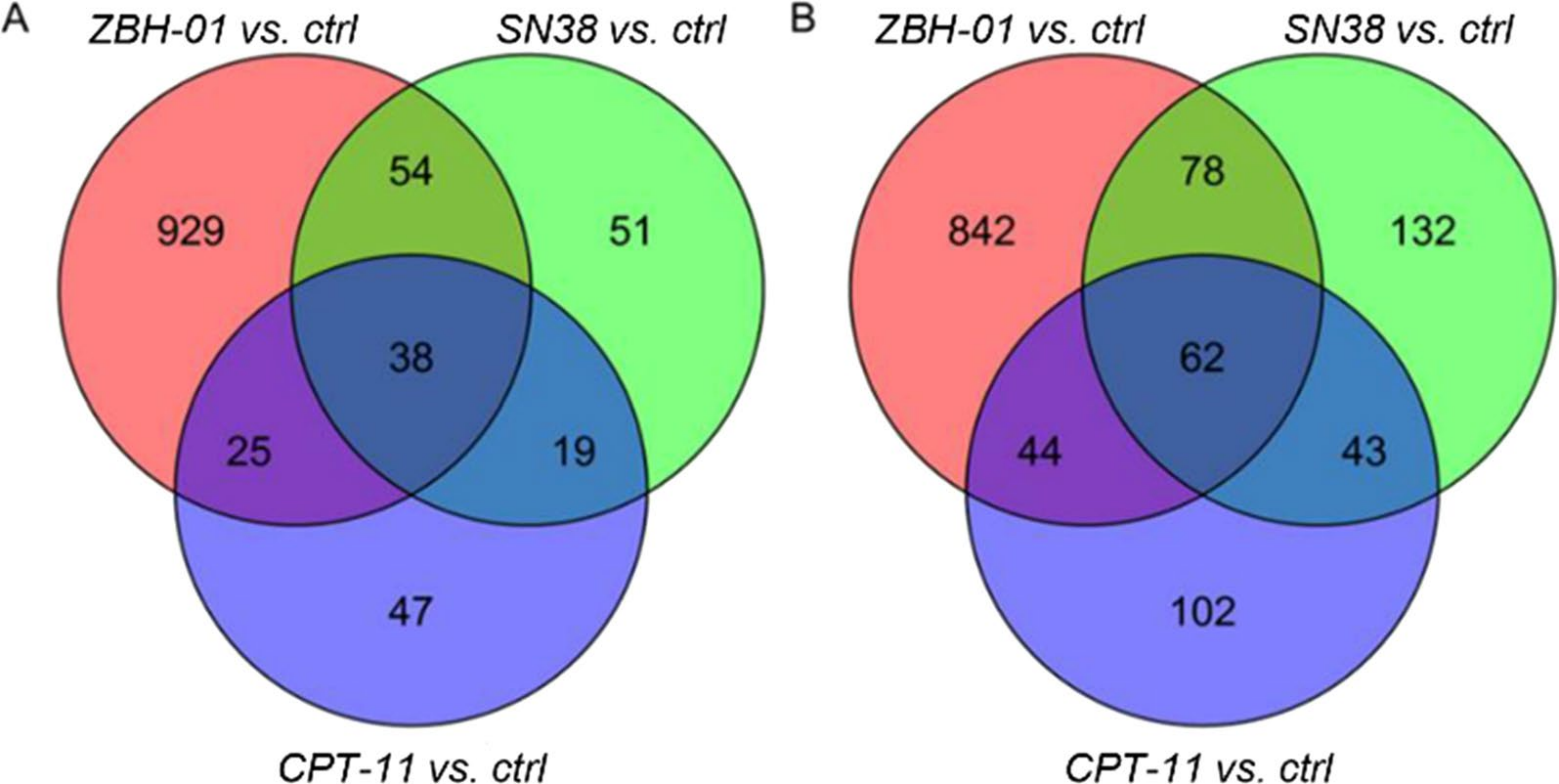
Venn diagrams for differentially expressed genes among ZBH-01, CPT-11, and SN38 groups. **A** Upregulated genes; **B** Downregulated genes

Previous studies have identified hundreds of abnormally expressed protein-coding genes in tumor cells after treatment with various drugs [21, 22]. Additionally, the gene expression profile of colon cancer cells can be used to predict and distinguish the response to multiple chemotherapeutic agents [23]. Hence, we used NGS to analyze expression changes in LS174T cells in response to ZBH-01 treatment. After performing the t-test, we used *p* < 0.05 and |Log_2_ fold change|> 1 as criteria to screen out 2072 DEmRNAs (1026 downregulated and 1046 upregulated) between ZBH-01 and controls; 380 DEmRNAs (251 downregulated and 129 upregulated) between CPT-11 and the control group; and 377 DEmRNAs (215 downregulated and 162 upregulated) between SN38 and controls. The Venn diagrams were used to classify the DEmRNAs among ZBH-01, CPT-11, and SN38 groups (Fig. 3A, B). The three groups shared 100 DEmRNAs (62 downregulated and 38 upregulated mRNAs). Compared to CPT-11 and SN38, the ZBH-01-treated group presented 1769 DEmRNAs (842 downregulated and 927 upregulated mRNAs). These results suggested that ZBH-01 might have a unique anti-tumor mechanism.

### Clustering of specific GO items and KEGG pathways in the ZBH-01-treated group

**Fig. 4:**
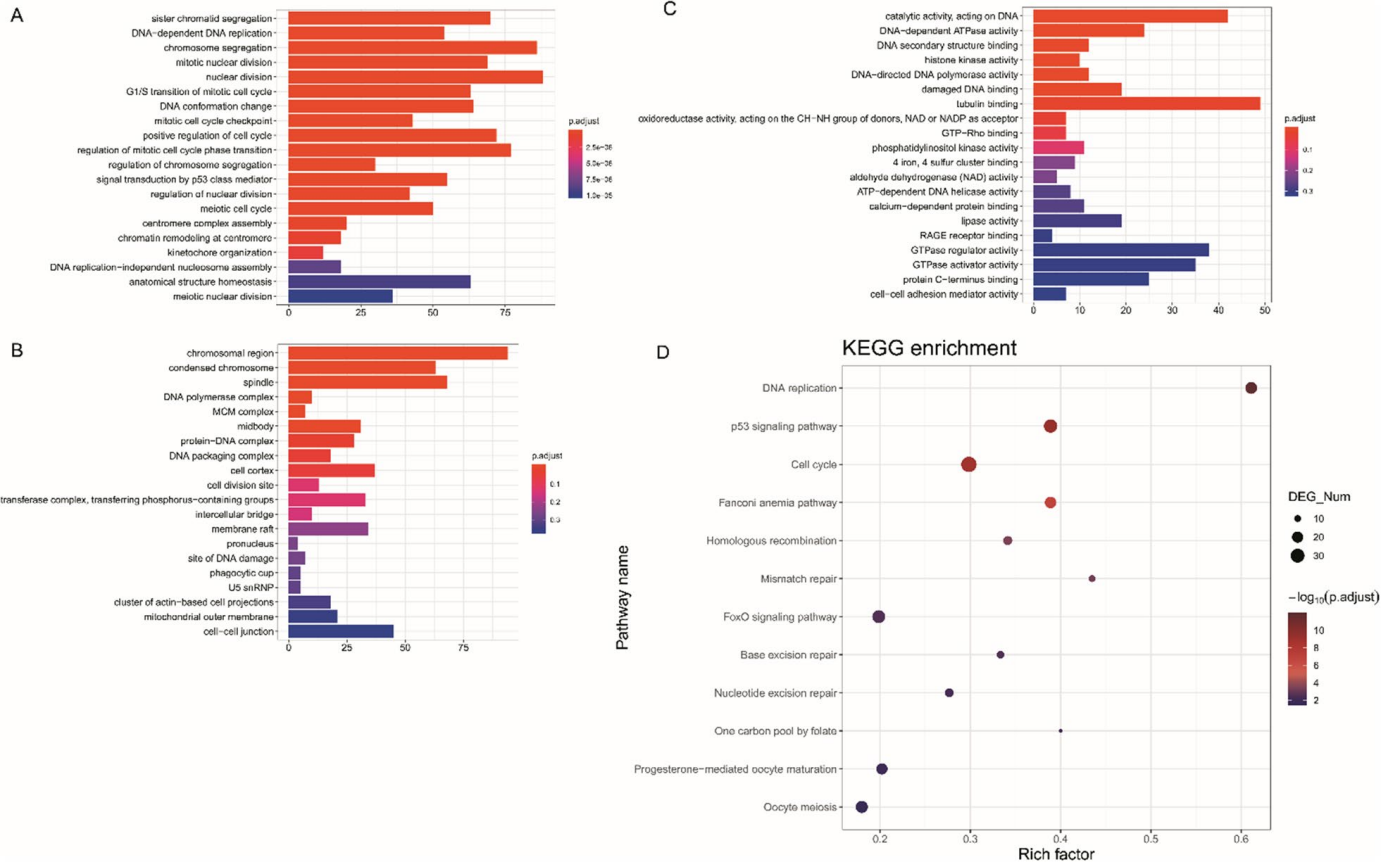
Significantly enriched GO terms and KEGG pathways for the DEmRNAs in the ZBH-01-treated group. **A** Biological processes (BP). **B** Cellular components (CC). **C** Molecular functions (MF). **D** KEGG pathways

Next, we performed GO enrichment analysis using 1769 DEmRNAs specific to the ZBH-01 group. The DEmRNAs were classified regarding their molecular functions (MF), biological processes (BP), and cellular components (CC). For BP (Fig. 4A), the DEmRNAs were mainly enriched in sister chromatid segregation, DNA-dependent DNA replication, and chromosome segregation. For CC (Fig. 4B), the DEmRNAs were mainly enriched in the chromosomal region, condensed chromosome, and spindle. Regarding MF (Fig. 4C), the DEmRNAs were mainly enriched in catalytic activity, acting on DNA, DNA-dependent ATPase activity, and DNA-secondary structure binding. The KEGG pathway results revealed that most ZBH-01 group-specific DEmRNAs were clustered in DNA replication, p53 signaling pathway, and cell cycle (Fig. 4D).

### The proteins encoded by DEmRNAs specific to the ZBH-01 group were prominently involved in cell cycle regulation

**Fig. 5:**
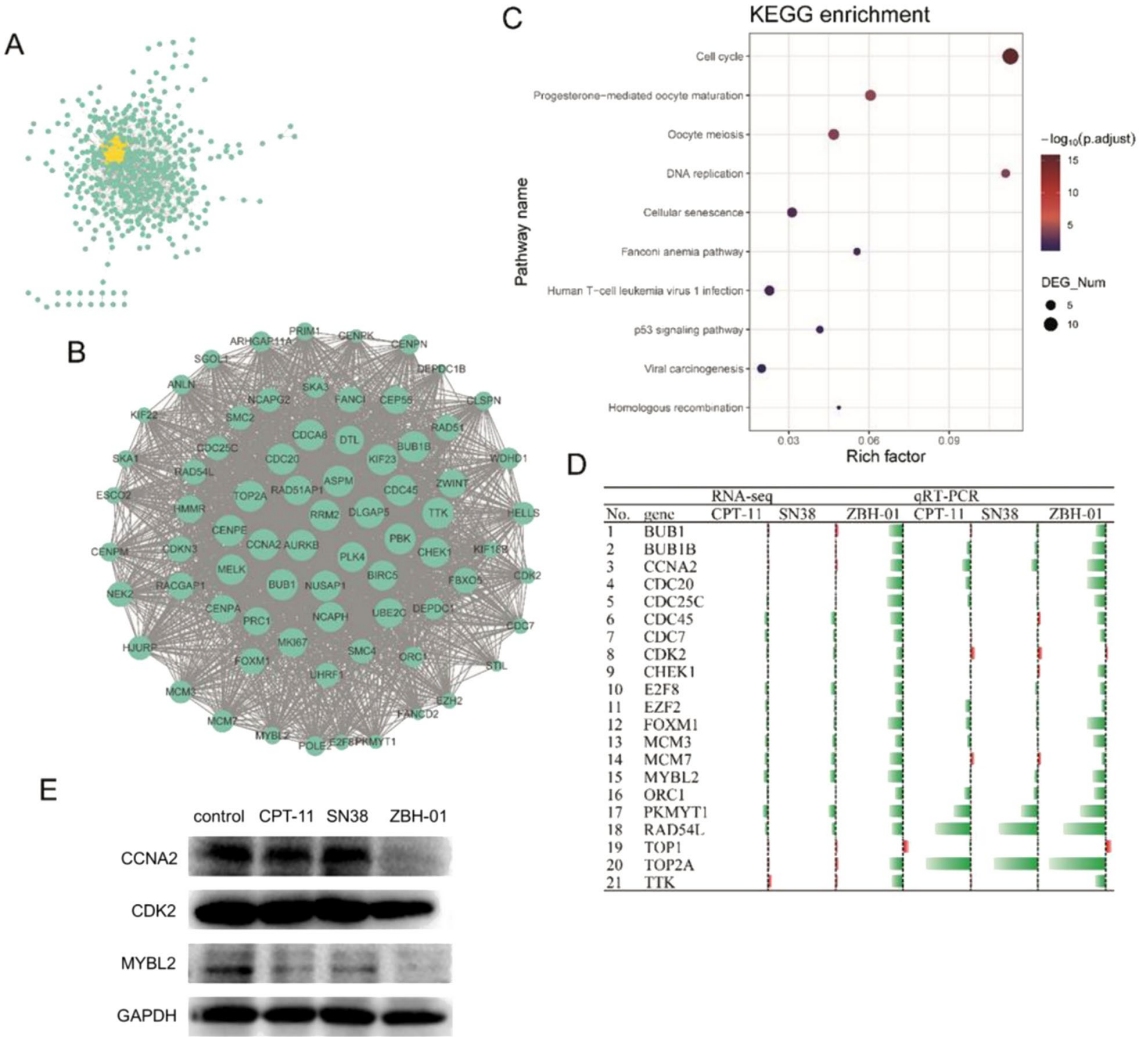
The proteins encoded by ZBH-01 group-specific DEmRNAs were prominently involved in cell cycle regulation **A**. The PPI network. The yellow part highlights the most prominent module. **B** Enlargement of the yellow module in (**A**), containing 73 genes. **C** Most significantly enriched KEGG pathway of these 73 genes. **D** qRT-PCR result showed the relative expression of some DEmRNAs in (**B**) in LS174T cells after treatment with 50 nmol/l ZBH-01, CPT-11, and SN38 24 h (logarithmic transformation). Green: downregulated; Red: upregulated. E, The result of western blot showed that ZBH-01 is more efficient than CPT-11 and SN38 in regulating the expression of three representative cell cycle related genes, MYBL2, CCNA2, and CDK2

We constructed a PPI network (Fig. 5A) based on the proteins encoded by ZBH-01 group-specific DEmRNAs using the STRING database (http://string-db.org) [24]. Then one prominent module (score = 61.833) were filtered out from the PPI network using Cytotype MCODE (Fig. 5B) [25]. This module consists of 73 downregulated genes. We conducted enrichment analysis again with these 73 genes demonstrating that they were principally associated with cell cycle, progesterone-mediated oocyte maturation, and oocyte meiosis. Fourteen genes (CDC45, CDC20, BUB1, CCNA2, BUB1B, TTK, CHEK1, CDC25C, MCM3, MCM7, ORC1, CDK2, CDC7, and PKMYT1) were involved in the most enriched cell cycle pathway (Fig. 5C). Their expression was further verified by qRT-PCR (Fig. 5D). It is noteworthy that among these 14 cell cycle-related genes, the interaction between transcription factor MYB proto-oncogene like 2 (MYBL2) and cyclin A2 (CCNA2)/cyclin dependent kinase 2 (CDK2) plays a decisive role in the cell cycle transition from the G_1_ to the S phase [26–32]. So we detected their expression by western blot. The result demonstrated that MYBL2, CCNA2, and CDK2 level were repressed in ZBH-01 group compared to CPT-11 and SN38 group (Fig. 5E). Whether these genes are the potential target of ZBH-01 and the more precise interaction between them during ZBH-01 treatment needs to be further studied.

Interestingly, we found that TOP2A is in the center of the module. In the relaxation assay results, we observed that the inhibition of ZBH-01 on TOP1 was weaker than CPT-11 and SN38. Thus, we need to further explore whether TOP2A is also a target of ZBH-01. Other key genes were also included in the module, such as FOXM1, RAD54L, UHRF1, MYBL2, EZH2, and E2F8, and play important roles in regulating the cell cycle.

### Relative expression of some ZBH-01 group-specific DEmRNAs by qRT-PCR

Next, we selected some genes in Fig. 5B to perform qRT-PCR. These genes included not only the aforementioned DEmRNAs, but also classical genes involved in the cell cycle, DNA replication, and apoptosis regulation because they play important roles in CPT-11 anti-tumor process as reported by our group and others [3, 6, 9, 11, 22]. The results demonstrated that the expression trend of most genes was consistent with the bioinformatics data and our previous report (Figs. 5D, 8).

### ***ZBH-01 induces G***_***0***_***/G***_***1***_***-phase arrest and increased apoptosis in colon cancer cells***

**Fig. 6:**
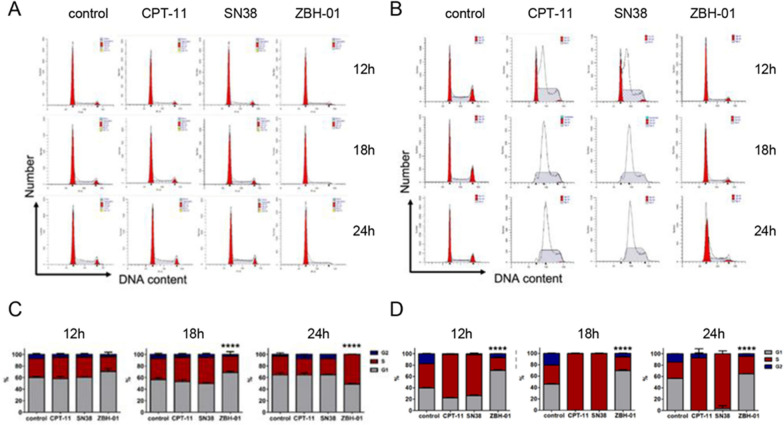
Cell cycle alteration in response to drug treatments. LS174T and SW1116 cells were treated with 50 nmol/l ZBH-01, CPT-11, and SN38 for 12, 18, and 24 h. The percentage of cells in G_0_/G_1_, S, and G_2_/M phases were analyzed by flow cytometry. **A** Histograms of cell cycle distribution of LS174T cells at different time points. **B** Statistical analysis of A. 12 h: χ^2^ = 7.5391,* p* = 0.2738; 18 h: χ^2^ = 40.6417, ^****^p < 0.0001; 24 h: χ^2^ = 60.8731, ^****^p < 0.0001 **C**. Histograms of cell cycle distribution of SW1116 cells at different times. **D** Statistical analysis of C. 12 h: χ^2^ = 96.9852, ^****^p < 0.0001; 18 h: χ^2^ = 242.4146, ^****^p < 0.0001; 24 h: χ^2^ = 197.4546, ^****^p < 0.0001. ANOVA & two-tailed t-test, n = 3

**Fig. 7:**
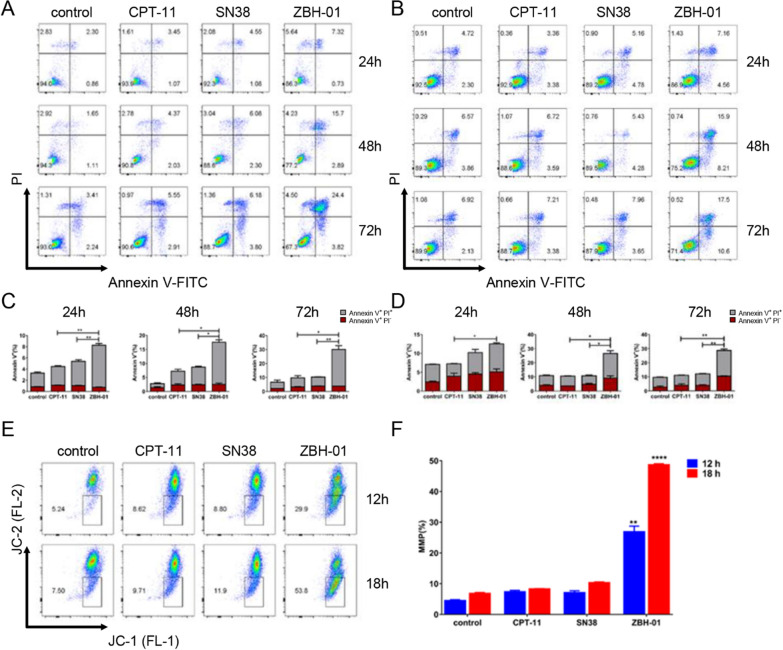
Induction of tumor cell apoptosis by ZBH-01, CPT-11, and SN38 (50 nmol/l, 24 h). **A** Scatter diagrams of apoptosis of SW1116 cells at different times. **B** Statistical analysis of A. Annexin V^+^/PI^−^ (%), 12 h: ^*^p = 0.0176, ZBH-01 vs. CPT-11. 48 h: ^*^p = 0.0284, ZBH-01 vs. CPT-11. 72 h: ^**^p = 0.0047, ZBH-01 vs. CPT-11; ^**^p = 0.0067, ZBH-01 vs. SN38. ANOVA & two-tailed t-test, n = 3. **C** Scatter diagrams of apoptosis of LS174T cells at different times. **D** Statistical analysis of C. Annexin V^+^/PI^−^ (%), 12 h: ^*^p = 0.0148, ZBH-01 vs. CPT-11; ^*^p = 0.0124, ZBH-01 vs. SN38. 18 h: ^*^p = 0.0341, ZBH-01 vs. CPT-11; ^*^p = 0.0374, ZBH-01 vs. SN38. 24 h: ^*^p = 0.0404, ZBH-01 vs. CPT-11; ^*^p = 0.0299, ZBH-01 vs. SN38. 48 h: ^*^p = 0.0399, ZBH-01 vs. SN38. 72 h: ^*^p = 0.0073, ZBH-01 vs. CPT-11; ^**^p = 0.0070, ZBH-01 vs. SN38. ANOVA & two-tailed t-test, n = 3. **E** Scatter diagrams of MMP of LS174T cells in response to drug treatment (50 nmol/l) at different time points. **F** Statistical analysis of **E**. 12 h: ^*^p = 0.0178, ZBH-01 vs. SN38. 18 h: ^**^p = 0.0047, ZBH-01 vs. CPT-11; ^**^p = 0.0047, ZBH-01 vs. SN38. 24 h: ^****^p < 0.0001, ZBH-01 vs. CPT-11; ^****^p < 0.0001, ZBH-01 vs. SN38. ANOVA & two-tailed t-test, n = 2

The above results showed that the gene expression features of LS174T cells treated with ZBH-01 were significantly altered compared to CPT-11 and SN38 groups. Most DEmRNAs were mainly involved in DNA replication, cell cycle, and P53 signal pathway. Thus, we evaluated the effects of ZBH-01 on the cell cycle and apoptosis by two colon cancer cell lines (LS174T and SW1116). The results showed that ZBH-01 preferably arrested tumor cells in the G_0_/G_1_ phase, while CPT-11 and SN38 arrested cells in the S phase (Fig. 6). Regarding apoptosis, ZBH-01 induced more apoptosis and necrosis than CPT-11 and SN38 (Fig. 7). These results are in accord with Fig. 5E. The more exact molecular mechanism is worthy of elucidation in the future.

**Fig. 8:**
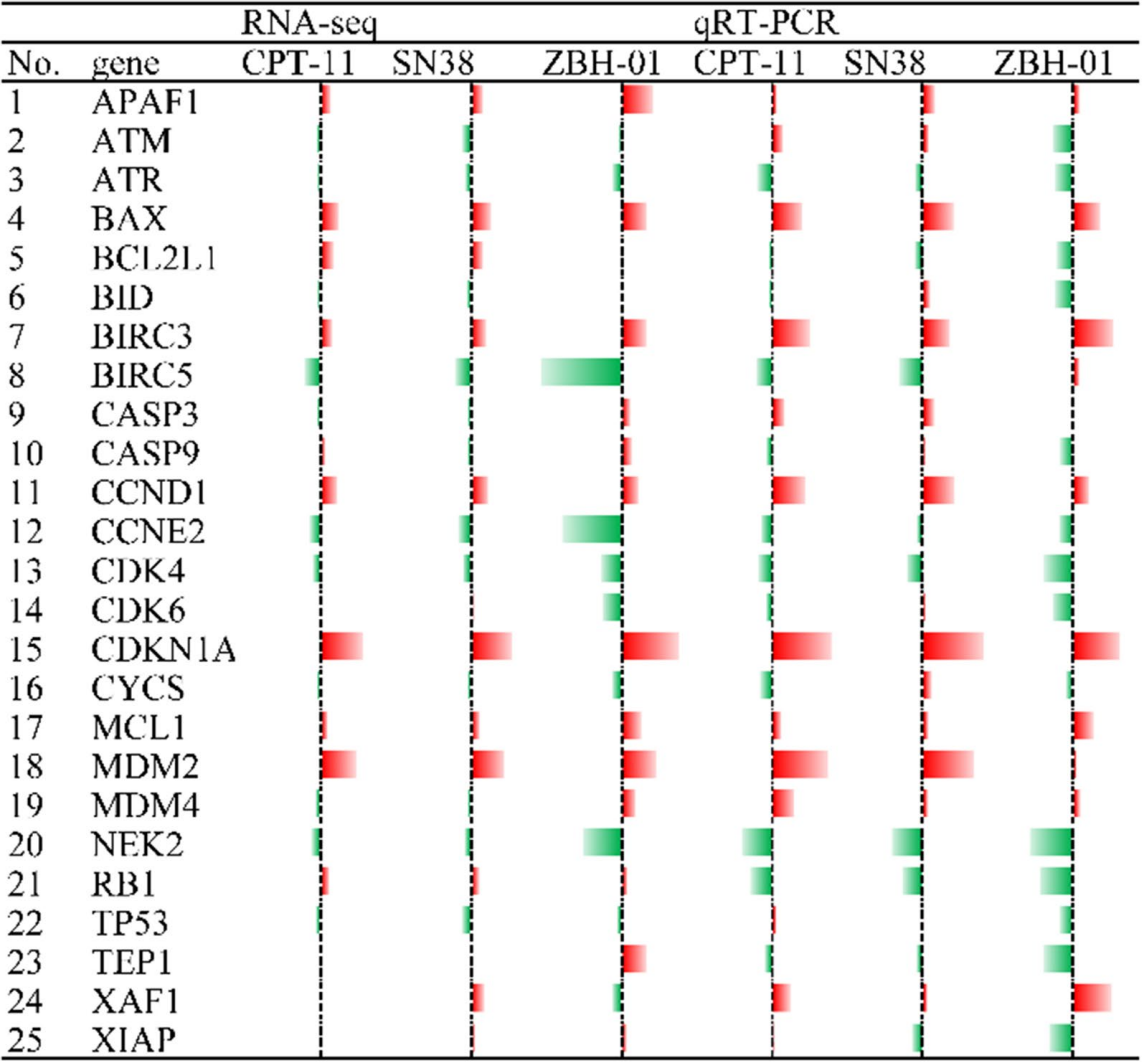
Relative mRNA expression of some differentially expressed genes involved in apoptosis in LS174T cells after treatment with 50 nmol/l ZBH-01, CPT-11, and SN38 24 h (logarithmic transformation). Green, downregulated; Red, upregulated

The cells’ ability to undergo apoptosis can be a major determinant of drug sensitivity [22]. Thus, to further analyze the effects of ZBH-01 on cell apoptosis, we detected the changes in mitochondrial membrane potential (MMP) in LS174T cells after drug treatment. The results showed that the MMP of the ZBH-01 group was significantly higher than CPT-11 and SN38 groups. We further verified the expression of some genes related to apoptosis using qRT-PCR, although they were not in the selected module of the PPI network. Some genes, including APAF1, ATR, BAX, BIRC3, CCND1, CCNE2, CDK4, CDK6, CDKN1A, MCL1, MDM2, and NEK2 showed consistent expression trends with the NGS result; while other genes, including ATM, BCL2L1, BID, BIRC5, CASP3, CASP9, CYCS, MDM4, RB1, TP53, TEP1, XAF1, and XIAP, displayed inconsistent expression trends (Fig. 8).

### ZBH-01 alters the expression of some key proteins related to cell apoptosis and DNA damage in colon cancer cells

**Fig. 9:**
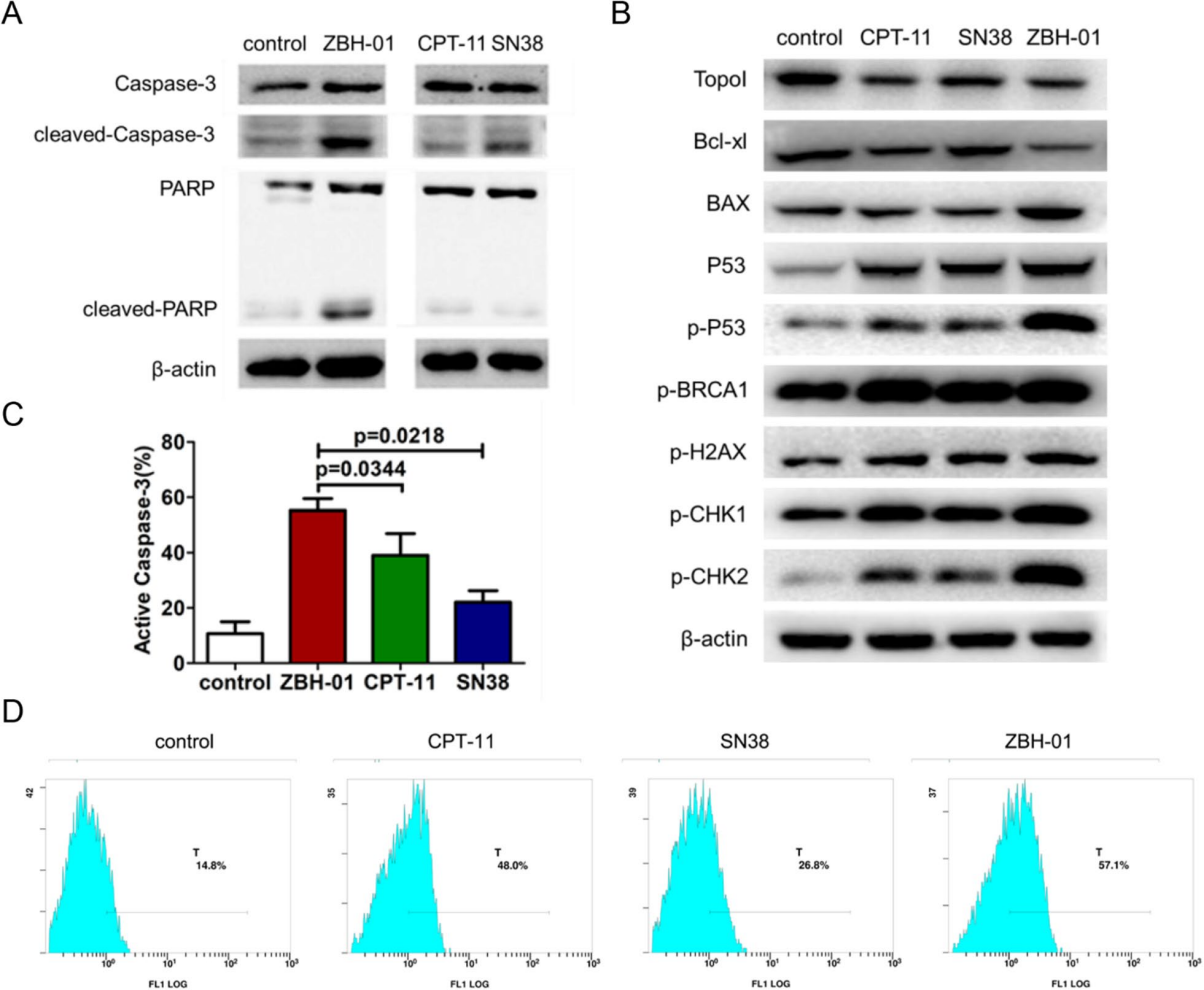
The alterative expression of some key proteins related to cell apoptosis and DNA damage in LS174T cells after treatment with ZBH-01, CPT-11, and SN38 (50 nmol/l, 24 h), respectively. **A**, **B** The result of western blot showed that ZBH-01 is more efficient than CPT-11 and SN38 in regulating the expression of representative proteins. **C** Histogram of statistical analysis showed the active caspase-3 level in LS174T cells after treatment with ZBH-01, CPT-11, and SN38 (50 nmol/l, 24 h), respectively. **D** Representative graph showed the active caspase-3 percentage detected by flow cytometry in LS174T cells. ANOVA & two-tailed t-test, n = 3

The aforementioned Fig 5E showed that ZBH-01 induced abnormal expression of several cell cycle-related genes. Here the expression changes of some key proteins related to apoptosis and DNA damage induced by ZBH-01 were similarly analyzed by western blot assay. The results showed that after treatment with 50 nmol/L ZBH-01, the level of cleaved-Cas3 and cleaved-PARP is both higher than that in CPT-11 and SN38 group (Fig 9A). CPT-11 and SN38 treatment exhibits comparable activity at the same condition. The Bax gene encodes a protein that promotes cell apoptosis, while another protein Bcl-xL can form heterodimer with other pro-apoptotic proteins to inhibit cell apoptosis. ZBH-01 presented higher effects on upregulation of Bax expression and downregulation of Bcl-xL level than CPT-11 and SN38 (Fig 9B). In addition, the level of activated caspase 3 in ZBH-01 group were significantly higher compare to CPT-11 and SN38 group (Fig 9C, D).

To characterize the DNA damage response during ZBH-01 treatment, the activation status and expression levels of proteins involved in DNA damage checkpoint pathways were measured in LS174T cells (Fig 9B). Among them, γ-H2AX and BRCA1 is the mediator of DNA damage, CHK1 and CHK2 is the transducer of DNA damage, and P53 is the effector of DNA damage [20]. The results showed that ZBH-01 treatment increased the expression of p-BRCA1, p-H2AX, p-CHK1, p-CHK2, and p-P53 as compare with CPT-11 and SN38, which indicates that ZBH-01 promises more efficient activatity in DNA damage. Altogether, these results suggested that ZBH-01 induced cell cycle arrest and apoptosis probably through generating more extent of DNA damage.

### *ZBH-01 represses tumor growth of colon cancer *in vivo*.*

**Fig. 10:**
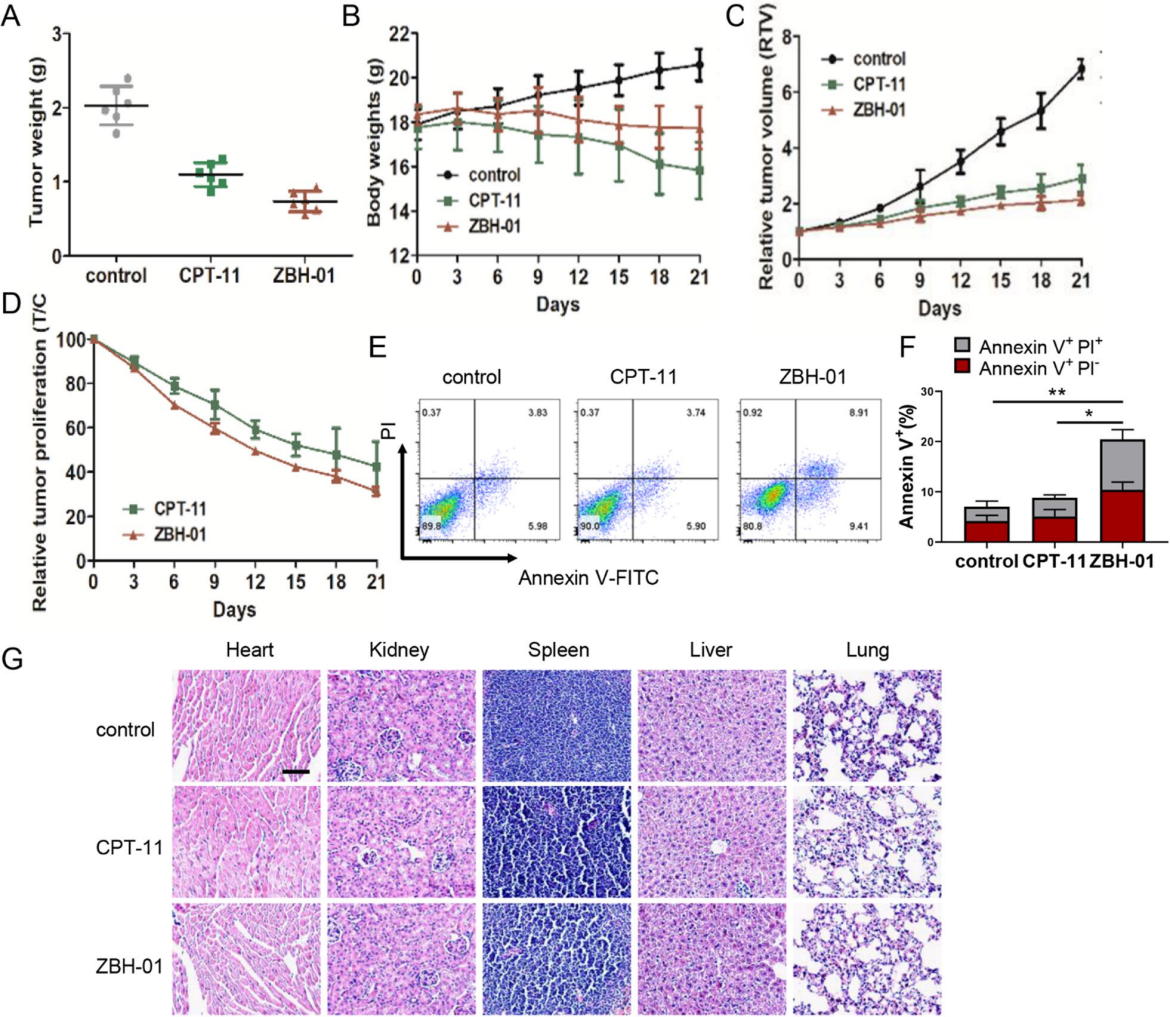
In vivo antitumor activity of ZBH-01 and CPT-11 in colon cancer xenograft model in nude mice. **A** Tumor weights (g) at the end of the experiment. ***p < 0.0001, CPT-11 vs. control, ***p < 0.0001, ZBH-01 vs. control, **p = 0.0019, ZBH-01 vs. CPT-11. **B** Body weights (g) at various times. ***p = 0.0001, CPT-11 vs. control, **p = 0.0040, ZBH-01 vs. control, **p = 0.0057, ZBH-01 vs. CPT-11. **C** Relative tumor volumes at various times. *p* = 0.0724, CPT-11 vs. control, *p = 0.0380, ZBH-01 vs. control, *p* = 0.2056, ZBH-01 vs. CPT-11. **D** Relative tumor proliferation at various time. *p* = 0.5030, ZBH-01 vs. CPT-11. ANOVA & t-test, two-tailed, n = 6. **E** The representative diagrams of cell apoptosis in vivo detected by flow cytometry. **F** Statistical analysis of E. **p = 0.0027, ZBH-01vs. control, *p = 0.0291, CPT-11 vs. control, ANOVA & t-test, two-tailed, n = 6. **G** Representative H&E staining images of the major organs (Heart, Kidney, Spleen, Liver, and Lung) in the control, CPT-11 and ZBH-01 treatment group. Scale bar = 20 µm

Xenografts models derived from LS174T cells were used to evaluate the antitumor effects of ZBH-01 and CPT-11 in vivo*.* Compared to controls, tumor growth was significantly suppressed in CPT-11- and ZBH-01-treated groups (Fig. 10A, C). The dosage of ZBH-01 seemed to cause a lighter loss of body weight than CPT-11, but was still significantly lower than the average weight of the control group (Fig. 10B). There was no significant difference in the rate of relative tumor proliferation between ZBH-01 and CPT-11 group (Fig. 10D). Nevertheless, the relative tumor volume was significantly lower in ZBH-01 group than in the control group, but not in the CPT-11 group. ZBH-01 induced a higher percentage of tumor cell apoptosis than CPT-11 (Fig. 10E, F). The results of H&E staining showed that ZBH-01 has good tolerable toxicities in vivo (Fig. 10G). The morphology and structures of the main organs, such as the heart, liver, spleen, lung, and kidney in ZBH-01-treated mice are all exhibited no apparent pathological abnormalities.

## Discussion

DNA topoisomerases, which can be divided into TOP1 and TOP2, are a class of enzymes that control the topological state of DNA through catalyzing the break and combination of DNA strands. Topoisomerase inhibitors are an important class of anti-tumor drugs since tumor cells have abnormally high expression of TOP1 and TOP2 [33]. Irinotecan (CPT-11) is a TOP1 inhibitor [34], which plays an important role in clinical treatment of metastatic colorectal cancer [35].

In the last three decades, many groups synthetized CPT-11 derivatives to enhance their cytotoxicity or minimize adverse events [18, 36, 37], but no new analogs have been approved so far. We synthesized a novel CPT-11 derivative ZBH-01 using a natural amino acid glycine group to replace the 4-piperidinopiperidine group to overcome the metabolism drawback of CPT-11. Then by conjugating the amino group of glycine to the 10-position of SN38 via a carbamate bond, the carboxyl group of ZBH-01 converted to sodium salt to improve its water solubility. ZBH-01 showed more potent antitumor activity in vitro even in the CPR-11-resistant cells. The 3D cell culture and in vivo xenograft model further confirmed superior anti-tumorigenesis effect of ZBH-01 than CPT-11 and SN38. ZBH-01 can rapidly convert to SN38 in both non-enzymatic physiological buffer (pH 7.4) and plasma in vitro. Its AChE inhibition activity and in vivo toxicity was also lower than that of CPT-11. All these characteristics of ZBH-01 might contribute to its antitumor potency.

We next compared the inhibitory effects of ZBH-01 and CPT-11/SN38 on TOP1 in colon cancer cells. The DNA relaxation assay unexpectedly showed that the inhibition of ZBH-01 on TOP1 was significantly lower than CPT-11 and SN38. This was inconsistent with our previous reports [9, 11]. However, the result of ICE bioassay showed that the inhibitory ability of ZBH-01 to TOP1 through chromatin-associated TOP cleavage complexes is higher than that of CPT-11 and SN38. We then confirmed this effect by NGS and qRT-PCR. Western blot result showed that TOP1 protein was slightly downregulated by ZBH-01, suggesting that ZBH-01 might inhibit TOP1 after transcription. Unexpectly, we observed that the TOP2A mRNA level were significantly repressed in the ZBH-01 group by NGS and qRT-PCR. This might be related to the chemical structure of ZBH-01. We intend to explore this issue in the future.

High-throughput-based gene expression profiling enables characterization of drug sensitivity of tumor cells [38] and identification of new drug targets [21]. We compared mRNA expression profiles of colon cancer cells treated with ZBH-01, CPT-11, and SN38, respectively. The results showed that ZBH-01 treatment remarkably induced a unique abnormal expression of 1769 DEmRNAs (842 downregulated and 927 upregulated mRNAs) in LS174T cells. These DEmRNAs were mainly enriched in DNA replication, p53 signaling pathway, and cell cycle. After filtered out one prominent module from the PPI network, we found that the 73 genes in the module mostly associated with the cell cycle. Interestingly, TOP2A was also located at the center of the module, which again reminds us of its importance.

**Fig. 11:**
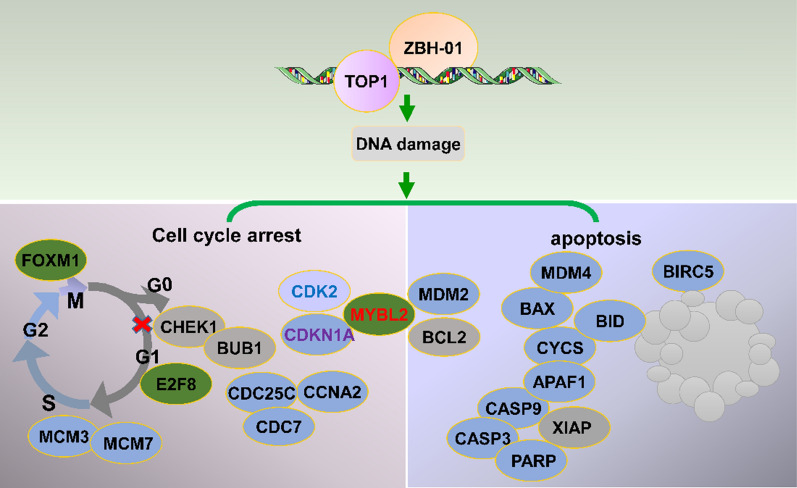
Hypothetical ZBH-01 mechanisms. The DNA damage caused by ZBH-01 induces not only cell cycle arrest but also apoptosis. CCNA2, CDK2, MYBL2, CHEK1, BAX, BCL2L1, caspase 3, and other genes might be regulated by ZBH-01

We then performed cell cycle and apoptosis assays to confirm the antitumor activity of ZBH-01. Under the same conditions (50 nmol/L, 24 h), ZBH-01 significantly induced more apoptosis and cell cycle arrest in the G_1_ phase in LS174T and SW1116 colon cancer cells; while CPT-11 and SN38 mainly induced cell cycle arrest in the S phase. Even after treatment 48 and 72 h, CPT-11 and SN38 did not induce apparent apoptosis. Furthermore, ZBH-01 presenting a stronger effect on inducing tumor cell apoptosis at 12 and 18 h earlier. These results were additionally verified by the MMP and western blot assay [39] and are consistent with others’ report that increasing expression of p53 after CPT treatment leading to cell cycle arrest [40], and CPT-11 inducing cell cycle arrest in the S- and G_2_/M-phases [20, 41]. γ-H2AX, BRCA1, CHK1, CHK2, and P53 are key regulators in various signaling pathways including DNA damage, cell cycle, and apoptosis [42]. Our results showed that ZBH-01 treatment increased the expression of p-BRCA1, p-H2AX, p-CHK1, p-CHK2, and p-P53 as compare with CPT-11 and SN38, which indicates that ZBH-01 promises more efficient activatity in DNA damage. Altogether, these results might explain that ZBH-01 induced more extent of DNA damage than CPT-11 and SN38. Subsequently, it facilitates cell cycle arrest by causing the interaction between CCNA2, CDK2, and MYBL2, and promotes cell apoptosis by regulating the expression of apoptosis-related genes (Fig. 11).

Our study has some limitations. First, we should analyze more tumor cell lines and other tumor models in mice. Second, we only verified the levels of some DEmRNAs by qRT-PCR. Neither the expressions of gene-encoded proteins nor the gain of or loss of function was evaluated. Furthermore, we did not add corresponding agonists or antagonists to help investigate the mechanisms of ZBH-01. Additionally, there are actual differences between the expression patterns of DEmRNAs in colon cancer tissues and cell lines after drug treatments, and how to fully elucidate these differences might be considered in the future.

Irinotecan has conspicuous advantages in the treatment of refractory metastatic colorectal cancer. It usually combined with other drugs in clinical scenes. AXEPT, a multicenter randomised phase III trial presented for the first time that the combination of capecitabine and irinotecan is effective and well tolerated in patients with advanced colorectal cancer [43]. CinClare is the first phase III trial using irinotecan combined with neoadjuvant radiochemotherapy for locally advanced rectal cancer treatment under the guidance of UGT1A1 genotype. The results show that the combined regimen with irinotecan significantly increased complete tumor response in Chinese patients [44]. Antibody drug conjugates (ADC) are a new type of biological drugs which conjugate monoclonal antibody and cytotoxic small molecule drugs through bioactive linkers [45]. Two FDA-approved ADCs, Enhertu (DS-8201) and Sacituzumab govitecan (IMMU132), are both designed based on irinotecan with SN38 as loading drug [46–48]. The new compound ZBH-01 was designed to reduce the severe side effects of irinotecan and improve it in vivo conversion efficiency to SN38. Theoretically, ZBH-01 has the potential to be used in any scenario where irinotecan can be applied. We need to investigate whether the combination of ZBH-01 and other drugs can improve the clinical response in the future.

## Data Availability

All data and materials related in this research are available for sharing.

## Abbreviation

BUB: BUB1 mitotic checkpoint serine/threonine kinase
BUB1B: BUB1 mitotic checkpoint serine/threonine kinase B
CCNA2: Cyclin A2
CDC20: Cell division cycle 20
CDC25C: Cell division cycle 25C
CDC45: Cell division cycle 45
CDC7: Cell division cycle 7
CDK2: Cyclin dependent kinase 2
CHEK1 (CHK1): Checkpoint kinase 1
E2F8: E2F transcription factor 8
EZH2: Enhancer of zeste 2 polycomb repressive complex 2 subunit
FOXM1: Forkhead box M1
MCM3: Minichromosome maintenance complex component 3
MCM7: Minichromosome maintenance complex component 7
MYBL2: MYB proto-oncogene like 2
ORC1: Origin recognition complex subunit 1
PKMYT1: Protein kinase, membrane associated tyrosine/threonine 1
RAD54L: RAD54 like
TOP1: DNA topoisomerase I
TOP2A: DNA topoisomerase II alpha
TTK: TTK protein kinase
APAF1: Apoptotic peptidase activating factor 1
ATM: Ataxia telangiectasia mutated
ATR: Ataxia telangiectasia related
BAX: BCL2 associated X, apoptosis regulator
BCL2L1: BCL2 like 1
BID: BH3 interacting domain death agonist
BIRC3 (c-IAP2): Baculoviral IAP repeat containing 3
BIRC5 (survivin): Baculoviral IAP repeat containing 5
CASP3: Caspase 3
CASP9: Caspase 9
CCND1: Cyclin D1
CCNE2: Cyclin E2
CDK4: Cyclin dependent kinase 4
CDK6: Cyclin dependent kinase 6
CDKN1A (P21): Cyclin dependent kinase inhibitor 1A
CYCS: Cytochrome c, somatic
MCL1: MCL1 apoptosis regulator, BCL2 family member
MDM2: MDM2 proto-oncogene
MDM4: MDM4 regulator of p53
NEK2: NIMA related kinase 2
RB1: RB transcriptional corepressor 1
TP53: Tumor protein p53
TEP1: Telomerase associated protein 1
XAF1: XIAP associated factor 1
XIAP (BIRC4): X-linked inhibitor of apoptosis
PARP: Poly-ADP-ribose-polymerase
